# Treatment of choroidal neovascularization in a case of factor VIII deficiency: Ten-year follow-up

**DOI:** 10.3205/oc000085

**Published:** 2018-02-22

**Authors:** Pukhraj Rishi, Ekta Rishi

**Affiliations:** 1 Shri Bhagwan Mahavir Vitreoretinal Services, Sankara Nethralaya, Chennai, India

## Abstract

An unusual case of choroidal neovascularization in a young female with factor VIII deficiency is presented. The treatment course lasted 23 months. A favourable treatment outcome with restoration of the original visual acuity was achieved. The eye remained stable until the last follow-up, 10 years following therapy.

## Case description

**Figure 1:**
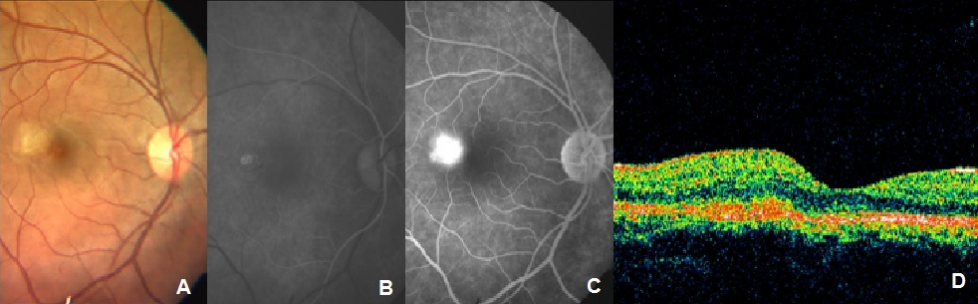
Color photo, early and late images of FFA and OCT picture at baseline

A 21-year-old female presented with complaints of seeing a black spot in front of the right eye associated with distortion of the central vision of one week duration. There was no history of trauma. Best-corrected visual acuity (BCVA) in both eyes was 20/20. Anterior segment examination was essentially normal in both eyes. Fundus examination of the right eye revealed a yellowish-gray subretinal lesion with subretinal fluid supero-temporal to the fovea and pigment epithelial detachment (Figure 1 (Fig. 1)). The lesion spared the fovea. Optic disc and retinal vasculature appeared normal and no vitreous cells were noted. Fundus examination of the left eye was normal. 

**Figure 2:**
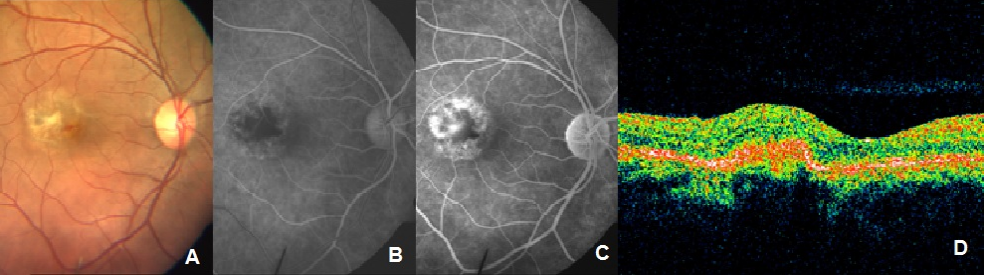
After PDT + IVTA. Persistent leakage from extrafoveal component, OCT shows absence of subretinal fluid.

The patient was a diagnosed case of factor VIII deficiency about a month back. Her coagulation profile and hemogram were normal. However, factor VIII – C level was 37.5% (Normal range 50–150%). Fundus fluorescein angiography (FFA) revealed a classic extrafoveal choroidal neovascular membrane (CNVM) (Figure 1 (Fig. 1)). Focal laser photocoagulation to the CNVM was done after discussing the treatment options with the patient. The patient reported after 6 weeks with visual acuity in the right eye 20/30, N6. Clinical examination revealed a regressing extrafoveal neovascular component and an active juxtafoveal neovascular component. Clinical findings were confirmed on FFA and optical coherence tomography (OCT) (Figure 2 (Fig. 2)). The patient was treated with photodynamic therapy (PDT) followed by intravitreal triamcinolone acetonide (IVTA) injection (4 mg/0.1 ml) 2 days later. After 6 weeks, visual acuity in the right eye was 20/30 and the intraocular pressure (IOP) was 32 mmHg. Topical Timolol maleate 0.5% twice a day was started. Since there was persistent hyperfluorescence related to the extrafoveal CNVM, thermal laser photocoagulation was done. 

With treatment, IOP came down to 12 mmHg in 2 weeks and the same treatment was continued. On the next visit eight weeks later, visual acuity was 20/60, N10. Clinical examination revealed recurrence of the juxtafoveal choroidal neovascular membrane, which was confirmed on FFA and OCT. The patient was treated with four intravitreal injections of bevacizumab (1.25 mg/0.05 ml) at an interval of 4–6 weeks. The IOP was found to have normalized and hence topical antiglaucoma treatment was stopped. 

**Figure 3:**
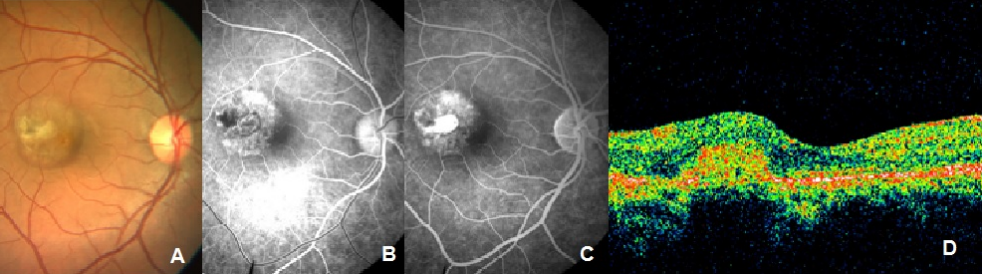
FFA and OCT findings after 4^th^ Injection of bevacizumab. FFA shows late leakage, no subretinal fluid detected on OCT.

**Figure 4:**
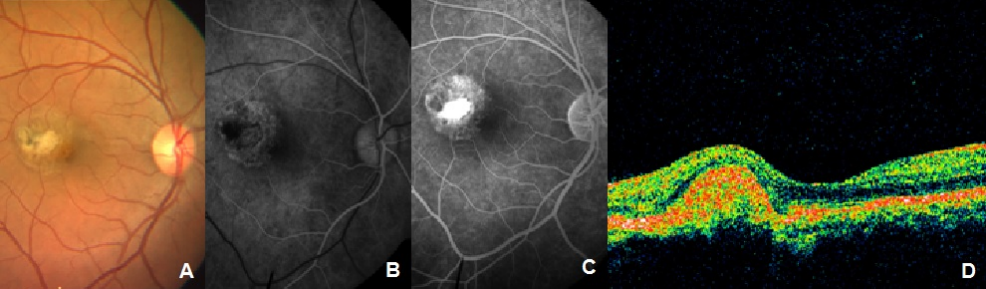
Findings on FFA and OCT at last visit. Staining of the scar is seen on FFA, no subretinal fluid visible on OCT.

Six weeks after the fourth injection, visual acuity in the right eye improved to 20/30, N6. CNVM appeared scarred (Figure 3 (Fig. 3)). However, there was persistent leakage on FFA. The patient was treated with a combination of PDT and intravitreal bevacizumab. After 7 weeks, visual acuity improved to 20/20. Clinically, CNVM appeared scarred. The patient was followed up at intervals of 2 weeks. Fourteen weeks later, the patient reported with an increase in distortion in the right eye. BCVA was 20/20 in the right eye. OCT showed subretinal fluid and FFA showed recurrent extrafoveal CNVM. The patient was treated with intravitreal bevacizumab (1.25 mg/0.05 ml). The patient was seen six weeks later when she reported resolution of symptoms; her visual acuity was 20/20. Clinical findings were confirmed on OCT and FFA (Figure 4 (Fig. 4)). When seen last (i.e. 10 years post bevacizumab injection), BCVA was maintained at 20/20 and the fundus remained stable. 

## Discussion

Various ophthalmic complications of coagulation disorders have been described. Rubenstein et al. reviewed 123 hemophiliacs, in which ocular complications such as subconjunctival hemorrhage, orbital or postoperative hemorrhage were noted in 25 patients [1]. Maguluri et al. reported delayed suprachoroidal hemorrhage in a patient of mild factor VIII deficiency [2]. Vitreous hemorrhage, pre- and subretinal hemorrhage have also been reported in 2 patients of von Willebrand’s factor deficiency [3]. Hereby we report a case of choroidal neovascularization and its treatment in a known patient of factor VIII deficiency that resolved after a protracted and varied treatment course with restoration of the original visual acuity. 

## Notes

### Competing interests

The authors declare that they have no competing interests.
